# Conjunctival repair after glaucoma drainage device exposure using collagen-glycosaminoglycane matrices

**DOI:** 10.1186/s12886-018-0721-6

**Published:** 2018-02-27

**Authors:** André Rosentreter, Alexandra Lappas, Randolf Alexander Widder, Maged Alnawaiseh, Thomas Stefan Dietlein

**Affiliations:** 1 Department of Ophthalmology, University of Würzburg, Josef-Schneider-Str. 11, 97080 Würzburg, Germany; 2 Center of Ophthalmology, University of Cologne, Cologne, Germany; 3 Department of Ophthalmology, St. Martinus-Krankenhaus Düsseldorf, Düsseldorf, Germany; 4 Department Of Ophthalmology, University of Muenster Medical Center, Muenster, Germany

**Keywords:** Episcleral drainage device, Baerveldt, Ahmed, Collagen-glycosaminoglycane matrix (CGM), Drainage tube, Conjunctival defect, Conjunctival repair, Conjunctival hole, Glaucoma drainage device, Biodegradable implant, Ologen implant

## Abstract

**Background:**

To report the results of the repair of conjunctival erosions resulting from glaucoma drainage device surgery using collagen-glycosaminoglycane matrices (CGM).

**Methods:**

Case series of 8 patients who underwent revision surgery due to conjunctival defects with exposed tubes through necrosis of the overlying scleral flap and conjunctiva after Baerveldt drainage device surgery. The defects were repaired by lateral displacement of the tube towards the sclera, with a slice of a CGM as a patch, covered by adjacent conjunctiva.

**Result:**

Successful, lasting closure (follow-up of 12 to 42 months) of the conjunctival defects was achieved without any side-effects or complications in all eight cases.

**Conclusions:**

Erosion of the drainage tube, creating buttonholes in the conjunctiva after implantation of glaucoma drainage devices, is a potentially serious problem. It can be managed successfully using a biodegradable CGM as a patch.

## Background

Conjunctival defects after penetrating glaucoma surgery, e.g. trabeculectomy or insertion of episcleral glaucoma drainage devices, are rare but severe complications. The defects often lead to leakage and contact between the anterior chamber and surrounding surfaces with the risk of subsequent carry-over of bacteria, blebitis and endophthalmitis [1–5].

In the case of trabeculectomy, especially after use of antimetabolites such as mitomycin-C (MMC) and 5-fluoruracile (5-FU), conjunctival defects can even occur a long time after surgery. Antimetabolites, which are very useful for prevention of scarring, affect wound healing processes and lead to the formation of thin-walled blebs [6].

After implantation of an episcleral glaucoma drainage device (GDD, e.g. the non-valved Baerveldt (Advanced Medical Optics, USA) or the valved Ahmed (New World Medical, USA) etc.), complications such as erosion of the tube (GDD-specific) or even the plate of the implant through the conjunctiva occur in 2–7% cases [1–5]. As with late bleb leakage after trabeculectomy, erosion of the conjunctiva exposing the tube of the GDD makes revision surgery necessary [7]. Erosion of the conjunctiva on top of the tube or the implant is more frequent in eyes with a history of multiple intraocular surgeries.

To repair erosion of the conjunctiva, a patch is usually placed on top of the tube and the conjunctiva is closed above this patch. Due to the surrounding scar tissue and the fragile structure of the frequently inflamed tissue around the tube, specialist knowledge and skill are required to achieve long-lasting wound closure in conjunctiva surgery. As reported in a case report [8], we favour a combination of re-fixation of the tube to the sclera with prior lateral displacement of the tube, followed by patching of the implant with a slice of a biodegradable implant (ologen™ implant, Aeon Astron Corporation, The Netherlands) in combination with conjunctival advancement. The aim of the lateral displacement of the tube is to avoid mechanical problems between the tube and lid margin at the previous point of conjunctival erosion, which could trigger repeated conjunctival erosion.

Since only a small number of patients suffer from conjunctival erosion after glaucoma drainage device surgery (2–7%) [1–5], we present a case-series of just eight patients who underwent such surgery. We hereby focus not only on the surgical success in covering the defect, but also on intraocular pressure, antiglaucomatous medication, visual acuity and the need for further surgical interventions.

## Methods

### Patients and preoperative examination

**Fig. 1:**
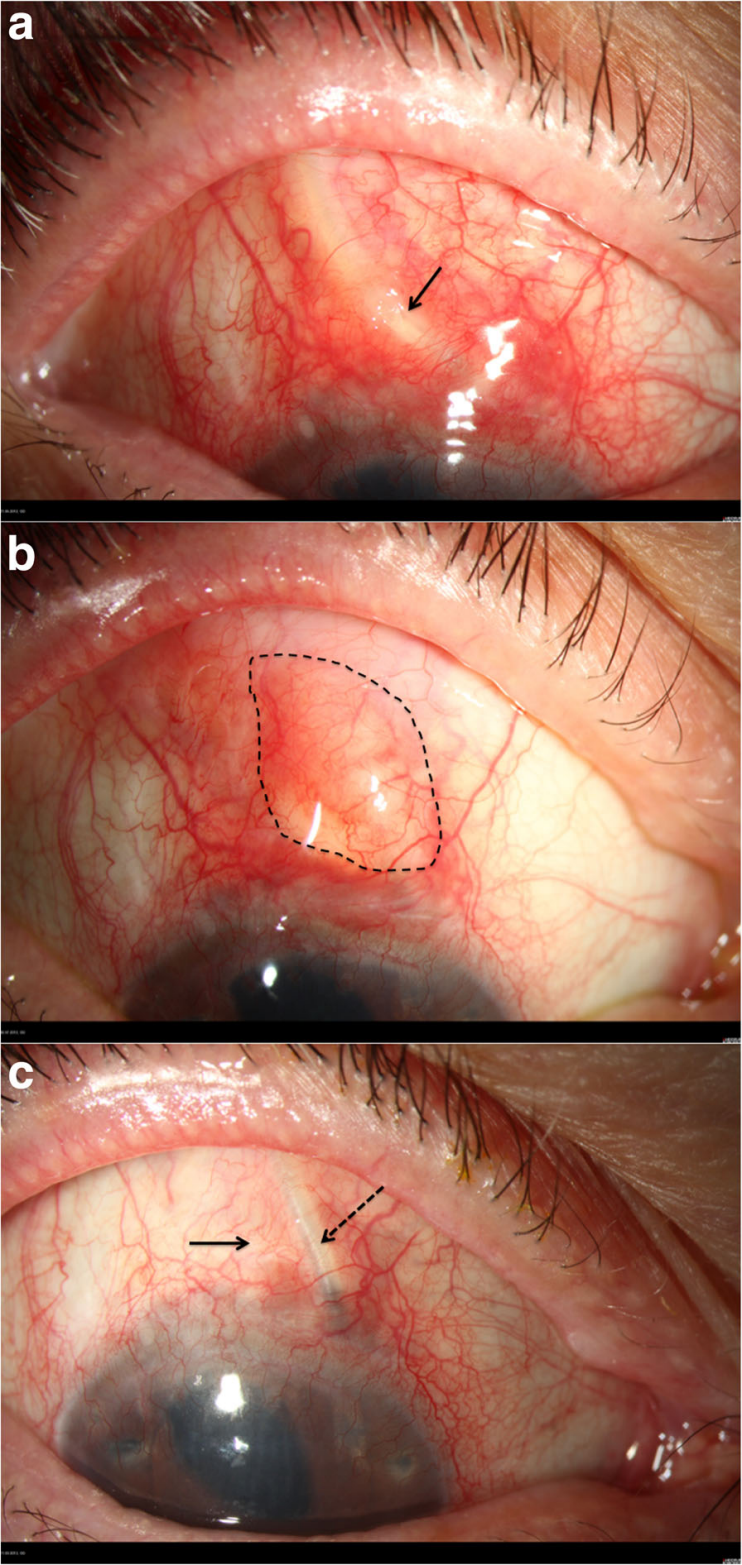
Chronological order. **a** Conjunctival buttonhole of 1 × 0.5 mm with exposure of the tube; arrow indicates the area of exposure. **b** Conjunctival defect closed with conjunctival hyperemia and underlying ologen implant at 2 months after surgery; dashed line shows the size of the ologen slice. **c** Stable re-epithelialisation at 10 months following surgery; arrow indicates the former area of exposure, the dashed arrow shows the displaced tube. The ologen implant has already been resorbed at this time (Complete resorption is usually seen between the third and sixth postoperative months)

Our study was based on a retrospective consecutive case series of eight patients who were treated for buttonholes [Fig. 1a] after glacuoma drainage device surgery between 2009 and 2016 in the Center of Ophthalmology, University of Cologne. Due to the retrospective study design and no further patient examinations, an ethics vote was considered unnecessary (§ 2 (1) and (2) of the Statutes of the Ethics Committee of the University of Lübeck). All surgeries were performed by an experienced glaucoma surgeon (TD). In all cases, an episcleral glaucoma drainage device was used after several preliminary operations (minimum 2) for intractable glaucoma. After a minimum of 1 month and a maximum of 6 years conjunctival erosion of the tube occurred.

Before surgical intervention, all patients underwent a baseline examination, which included measurement of best-corrected visual acuity (ETDRS charts, Lighthouse, Long Island, USA), visual field examination (30–2, Octopus perimeter 101, Haag-Streit, Switzerland), biomicroscopy, gonioscopy, and Goldmann applanation tonometry.

### Surgical technique and follow-up

**Fig. 2:**
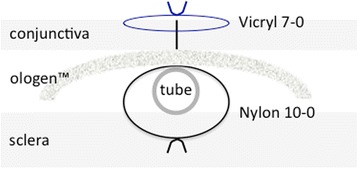
Schematic view of the different layers at the end of surgery (GDD: glaucoma drainage device)

The surgical procedure was performed under either general or local anesthesia, according to the patient’s preference. As described in our previous publication [8], the tubes were laterally displaced, fixed to the sclera with a horizontal mattress 10–0 nylon suture (knots recessed to the sclera), and covered with a slice of an ologen™ implant of 1–2 mm thickness. The conjunctiva was closed after mobilisation with a rotational flap of adjacent conjunctiva [Fig. 1b & c; 2]. Postoperatively, the patients received topical antibiotics three times a day for 2 weeks and low-dose steroids three times a day for 3 weeks.

Postoperative examinations were performed on a daily basis during hospitalisation. After hospitalisation, follow-up visits were arranged at 1 and 4 weeks and 3, 6, 12 and 24 months after surgery. At each visit all the above-mentioned examinations, except for visual-field testing and gonioscopy, were repeated. Side-effects and complications were recorded during postoperative follow-ups.

### Ologen™ implant

The ologen™ implant (Aeon Astron Europe B.V., the Netherlands) is a porous implant comprising > 90% lyophilized porcine atelocollagen and < 10% lyophilized glycosaminoglycan with a pore size of 20 to 200 μm [Fig. 2]. In our study we used a cylindrical (12 mm diameter) implant of 1 mm in height, either folded or unfolded, and in some cases cut down to a slice of 5 × 5 mm.

Atelocollagen is a highly purified pepsin-treated type I collagen. A collagen molecule has an amino acid sequence, known as a telopeptide, at both N- and C-terminals, which confers most of the collagen’s antigenicity. Atelocollagen obtained by pepsin treatment is low in immunogenicity, because it is free of telopeptides [8, 9].

### Statistics

Primary endpoint and surgical success were defined as complete wound closure without leakage of aqueous humour. Secondary endpoints were IOP control without the need for further revision surgery. Pre- and postoperative antiglaucomatous medication was classified according to a medication score [10].

The datasets used and/or analysed during the current study available are from the corresponding author on reasonable request. Statistical analysis was performed using Prism software (version 5, GraphPad software). Differences between preoperative and postoperative IOP and medication were compared by the non-parametric *t*-test, as the values were considered to be distributed non-parametrically (Mann-Whitney test, two-tailed). *P*-values of less than 0.05 were considered statistically significant.

## Results

In most cases the buttonholes occurred directly adjacent to the lid margin. Patient characteristics are given in Table 1; showing that the patients had multiple preoperated eyes. Erosion of conjunctiva occurred after a minimum of one and a maximum of 70 months after glaucoma drainage device surgery with a mean of 25.1 ± 30.8 months. We could not detect any ologen™ specific side-effects such as allergy or translocation of the implant on postoperative follow-up visits. No severe postoperative complications were detected and conjunctiva remained healthy and closed on follow-up.

**Table 1:** Patient characteristics for the presented case series of eight patients

#	Age [years]	Type of glaucoma	Preliminary surgeries	Erosion of conjunctiva after *xxx* months	BCVA [LogMAR]
P1	51	Angle closure	IE, Phaco, ppV, TE	70	0.20
P2	49	XFG	Phaco, TA, TE	2	0.20
P3	12	Uveitic	Phaco, TE	1	0.20
P4	57	Silicon oil	ppV, TE	4	0.80
P5	41	Silicon oil	ppV, TE	4	1.00
P6	81	Uveitic	Phaco, TE	60	Light perception
P7	18	Uveitic	Phaco, ppV	56	0.60
P8	44	Traumatic	2× TE	4	0.00
Mean ± SD	44.1 ± 21.8			25.1 ± 30.8	

*BCVA* best-corrected visual acuity, *IE* Iridectomy, *Phaco* Phacoemulsification, *ppV* pars plana vitrectomy, *SD* standard deviation, *TA* trabecular aspiration, *TE* trabeculectomy, *XFG* Pseudoexfoliation glaucoma

The course of intraocular pressure changes and medication score is shown in Table 2. The mean follow-up time was 26.8 ± 9.0 months. Further glaucoma surgery was necessary in three cases: in one case (patient #2) an additional glaucoma drainage device was used, while in two cases (patient #4 & #6) additional cyclophotocoagulation was applied.

**Table 2:** Intraocular pressure and antiglaucomatous medication score preoperative and at different points of follow-up

Intraocular pressure [mmHg]				
#	preOP	d1	12 m	Last follow-up (xx mo)
P1	15	13	15	16 (24 mo)
P2	19	23	15	13 (24 mo)
P3	25	10	12	14 (24 mo)
P4	17	–	15	9 (42 mo)
P5	25	24	17	16 (24 mo)
P6	19	11	19	19 (12 mo)
P7	16	21	6	9 (28 mo)
P8	14	–	12	19 (36 mo)
Mean ± SD	18.8 ± 4.2	17.0 ± 6,4	13.9 ± 3.9	14.4 ± 3.9
Medication score				
#	preOP	d1	12 m	Last follow-up (xx mo)
P1	2	2	0	0 (24 mo)
P2	0	0	6	0 (24 mo)
P3	10	0	5	4 (24 mo)
P4	6	–	6	0 (42 mo)
P5	8	5	5	5 (24 mo)
P6	0	0	6	6 (12 mo)
P7	0	0	0	0 (28 mo)
P8	3	–	3	4 (36 mo)
Mean ± SD	3.6 ± 3.9	1.2 ± 2.0	3.9 ± 2.6	2.4 ± 2.6

12 m = 12 months after surgery; d1 = 1 day after surgery; IOP = intraocular pressure; MedScore = medication score; preOP = preoperative

## Discussion

Glaucoma drainage device surgery is a useful adjunct in the treatment of refractory glaucoma despite having a few serious complications [1, 11–13]. As a consequence of the results of the TVT study [14, 15], glaucoma drainage device surgery is now being used more frequently than in the past and at an earlier stage (typical situation: Pseudophakic eyes with one or two failed trabeculectomies). Since this surgical method is now carried out more frequently, an increasing number of typical side-effects should be taken into consideration. One such complication is thinning of the conjunctiva directly above the tube with complete erosion of the overlying tissue [5]. This may result in leakage and is always an indication for revision surgery, as this entrance gate is a potential source of endophthalmitis [7].

The exact mechanism of conjunctival erosion remains unclear since, as far as could be seen, the implants and the tubes were correctly positioned. The buttonholes are probably formed either due to mechanical stress at the lid margin or due to the gap between the tube and sclera posterior to the scleral flap arising through the architecture of the glaucoma drainage devices near the basal plate.

To prevent conjunctival erosion, the tube should be patched during primary surgery and the patch covered with conjunctiva. The tube can be patched either with a scleral flap or with bovine pericardium, human sclera or other materials. The use of patched tubes has decreased the exposure rate from 30% to less than 5% [5]. In our case series all tubes were patched with a scleral flap in primary surgery.

Should a penetrating defect in the conjunctiva nevertheless occur during follow-up, it can be difficult to achieve closure of conjunctiva. A few methods for closing these defects in the conjunctiva have been described previously: For direct suture of the defect and use of conjunctival autografts, the results in literature show different success rates: A simple conjunctival closure is inadequate [16]. Reports on conjunctival autografts and conjunctival closure with a patch graft provided better results (Success rates: acellular human dermis patch graft: 83%; autologous scleral lamellar graft: 100%) [5, 16]. Also good results have been reported with amniotic membranes and additional application of autologous serum in a few cases of conjunctival erosion (Three cases; 100% success; follow-up: 6–30 months) [17].

Preliminary studies on bioengineered, biodegradable implants suggest that a porous collagen-glycosaminoglycane matrix (CGM; ologen™) will reduce conjunctival contraction and promote formation of an almost normal subconjunctival stroma [18–20]. Moreover, the use of the ologen™ implant was also described in primary glaucoma drainage device surgery as a patch with a good success rate [21]. This led us to try CGM in a single case of revision surgery after buttonhole formation on top of the drainage device tube [8]. After use of a total of eight CGM in revision surgery, we can confirm the persistent closure of conjunctival defects in all cases. No severe postoperative complications or CGM-specific side-effects were detected in our study series.

Except the patching of the tube with an ologen™ implant, the lateral displacement of the tube also appears to be decisive in our approach. This prevents the previously damaged conjunctiva from contact with the tube.

## Conclusion

Our case series shows that the described method with lateral displacement of the tube and patching with CGM is a possible alternative in revision surgery for repair of eroded conjunctiva overlying the tube of the glaucoma drainage device. Possible positive effects of the CGM are the avoidance of direct contact between the conjuctiva and the tube and the fact that CGM acts as a wound healing scaffold for structurally more normal tissue rather than structurally deficient scar tissue. Moreover, CGM probably minimizes movement of the conjunctiva in relation to the tube thus facilitating wound healing.

## Abbreviations

5-FU: 5-fluoruracile
CGM: Collagen-glycosaminoglycane matrices
GDD: Glaucoma drainage device
IOP: Intraocular pressure
MMC: Mitomycin-C
TVT study: Tube versus trabecuectomy study

## References

[1] Ayyala RS, Zurakowski D, Smith JA, Monshizadeh R, Netland PA, Richards DW (1998). A clinical study of the Ahmed glaucoma valve implant in advanced glaucoma. Ophthalmology.

[2] Lim KS, Allan BDS, Lloyd AW, Muir A, Khaw PT (1998). Glaucoma drainage devices; past, present, and future. Br J Ophthalmol.

[3] Aslanides IM, Spaeth GL, Schmidt CM, Lanzl IM, Gandham SB (1999). Autologous patch graft in tube shunt surgery. J Glaucoma.

[4] Siegner SW, Netland PA, Urban RC, Williams AS, Richards DW, Latina MA (1995). Clinical experience with the Baerveldt glaucoma drainage implant. Ophthalmology.

[5] Heuer DK, Budenz D, Coleman A (2001). Aqueous shunt tube erosion. J Glaucoma.

[6] DeBry PW, Perkins TW, Heatley G, Kaufman P, Brumback LC (2002). Incidence of late-onset bleb-related complications following trabeculectomy with mitomycin. Arch Ophthalmol.

[7] Francis BA, DiLoreto DA, Chong LP, Rao N (2003). Late-onset bacteria endophthalmitis following glaucoma drainage implantation. Ophthalmic Surg Lasers Imaging.

[8] Rosentreter A, Schild AM, Dinslage S, Dietlein TS (2012). Biodegradable implant for tissue repair after glaucoma drainage device surgery. J Glaucoma.

[9] Stenzel KH, Miyata T, Rubin AL (1974). Collagen as a biomaterial. Annu Rev Biophys Bioeng.

[10] Jacobi PC, Krieglstein GK (1995). Trabecular aspiration. A new mode to treat pseudoexfoliation glaucoma. Invest Ophthalmol Vis Sci.

[11] Goulet RJ, Phan AD, Cantor LB, WuDunn D (2008). Efficacy of the Ahmed S2 glaucoma valve compared with the Baerveldt 250 mm2 glaucoma implant. Ophthalmology.

[12] Syed HM, Law SK, Nam SH, Li G, Caprioli J, Coleman A (2004). Baerveldt-350 implant versus Ahmed valve for refractory glaucoma. A case-controlled comparison. J Glaucoma.

[13] WuDunn D, Phan AD, Cantor LB, Lind JT, Cortes A, Wu B (2006). Clinical experience with the Baerveldt 250 mm2 glaucoma implant. Ophthalmology.

[14] Gedde SJ, Schiffman JC, Feuer WJ, Herndon LW, Brandt JD, Budenz DL, Tube Versus Trabeculectomy Study Group (2012). Treatment outcomes in the tube versus trabeculectomy (TVT) study after five years of follow-up. Am J Ophthalmol.

[15] Gedde SJ, Herndon LW, Brandt JD, Budenz DL, Feuer WJ, Schiffman JC, Tube Versus Trabeculectomy Study Group (2012). Postoperative complications in the tube versus trabeculectomy (TVT) study during five years of follow-up. Am J Ophthalmol.

[16] Kalenak JW (2010). Revision for exposed anterior segment tubes. J Glaucoma.

[17] Ainsworth G, Rotchford A, Dua HS, King AJ (2006). A novel use of amniotic membrane in the management of tube exposure following glaucoma tube shunt surgery. Br J Ophthalmol.

[18] Chen HS, Ritch R, Krupin T, Hsu WC (2006). Control of filtering bleb structure through tissue bioengineering: an animal model. Invest Ohthalmol Vis Sci.

[19] Hsu WC, Ritch R, Krupin T, Chen HS (2008). Tissue bioengineering for surgical bleb defects: an animal study. Graefes Arch Clin Exp Ophthalmol.

[20] Hsu WC, Spilker MH, Yannas IV, Rubin PA (2000). Inhibition of conjunctival scarring and contraction by a porous collagen-glycosaminoglycan implant. Invest Ophthalmol Vis Sci.

[21] Stephens JD, Sarkisian SR (2016). The use of collagen matrix (Ologen) as a patch graft in glaucoma tube shunt surgery, a retrospective chart review. F1000Res.

